# Femtosecond laser induced thermophoretic writing of waveguides in silicate glass

**DOI:** 10.1038/s41598-021-87765-z

**Published:** 2021-04-16

**Authors:** Manuel Macias-Montero, Francisco Muñoz, Belén Sotillo, Jesús del Hoyo, Rocío Ariza, Paloma Fernandez, Jan Siegel, Javier Solis

**Affiliations:** 1 Laser Processing Group, Institute of Optics (IO, CSIC), Serrano 121, 28006 Madrid, Spain; 2 Institute of Ceramics and Glass (ICV, CSIC), Kelsen 5, 28049 Madrid, Spain; 3 Department of Materials Physics, Faculty of Physics, University Complutense of Madrid, 28040 Madrid, Spain; 4 Department of Optics, Faculty of Physics, University Complutense of Madrid, 28040 Madrid, Spain

**Keywords:** Materials science, Optics and photonics, Physics

## Abstract

Here in, the fs-laser induced thermophoretic writing of microstructures in ad-hoc compositionally designed silicate glasses and their application as infrared optical waveguides is reported. The glass modification mechanism mimics the elemental thermal diffusion occurring in basaltic liquids at the Earth’s mantle, but in a much shorter time scale (10^8^ times faster) and over a well-defined micrometric volume. The precise addition of BaO, Na_2_O and K_2_O to the silicate glass enables the creation of positive refractive index contrast upon fs-laser irradiation. The influence of the focal volume and the induced temperature gradient is thoroughly analyzed, leading to a variety of structures with refractive index contrasts as high as 2.5 × 10^–2^. Two independent methods, namely near field measurements and electronic polarizability analysis, confirm the magnitude of the refractive index on the modified regions. Additionally, the functionality of the microstructures as waveguides is further optimized by lowering their propagation losses, enabling their implementation in a wide range of photonic devices.

## Introduction

Fs-laser writing is a versatile tool to produce multipurpose photonic devices in glass^1^. Its effectiveness relies on strong-field ionization to produce modification in otherwise transparent dielectric materials^2–5^. When a fs-laser beam is tightly focused inside a glass, a large amount of energy is absorbed, rendering in a high local temperature increase on the order of 10^4^ K^6,7^. In such conditions, thermophoresis plays a key role in the final relative concentration of each element composing the glass^8^. Since the optical properties of a glass, such as refractive index or light absorption, strongly depend on its composition^9,10^, controlling the thermophoretic migration would enable the design of optically adjustable materials. This possibility has fostered investigations on the fundamentals of this phenomenon and its application in the field of photonics through local modifications of composition^11,12^.

Previous studies on high-repetition rate fs-laser induced element redistribution in glasses argue that the underlying mechanism is the result of a high temperature viscoelastic deformation and compressive stress induced by thermal expansion and boundary strains^8,12,13^. Indeed, thermophoresis threshold temperature is related to the viscosity of the glass, which may change up to twelve orders of magnitude from the glass transition range to the melt state^8,12^. However, the majority of studies focus their attention on the maximum temperature achieved, barely addressing the influence of another key parameter in thermal migration, the temperature gradient. Classical Soret effect studies on liquid silicates are based on such temperature falls and are capable of obtaining sensible information, such as the relative concentration of oxygen isotopes that can be linked to basaltic liquids of metasomatic veins in the Earth’s mantle^14^. Although time scales are substantially different, we would like to stress the importance of this parameter that could provide further insights on the fs-laser induced thermal migration mechanism.

Another important aspect to consider is the morphology of the modified regions, in terms of shape and size of the area subjected to compositional redistribution. In this sense, Kazansky et al*.* showed the existence of Quill laser writing in beams with intentionally tilted pulse fronts and the formation of so called flower and geyser shaped structures^15,16^. Alternatively, multipoint simultaneous irradiations carried out by Sakakura et al*.* demonstrated the possibility of creating a ribbon-shaped Si-rich glass^17^. In any case, the geometry of the modified area needs to be carefully studied since it plays a fundamental role in applications such as waveguides or optical amplifiers^12^.

A common feature in static fs-laser induced element redistribution studies is the use of standard or commercial glasses, such as soda-lime glass or BK7^17,18^. Although these glasses are abundant and economical, their compositions cause difficulties for creating high performance photonic devices. For this reason, we have initiated a long-term research project to design and fabricate fs-laser writable phosphate, borate and now silicate glasses^19,20^. A key feature is to modify the glass composition by adding small amounts of heavy oxides, such as BaO, a naturally abundant and cost-effective material^21^. The combination of these heavy with other lighter modifiers (e.g., Na_2_O, K_2_O) enables us to produce local positive refractive index increments upon fs-laser irradiation^19^. Additionally, scanning the sample through the beam leads to the creation of continuous smooth linear structures with high performance as infrared optical waveguides^22^. Furthermore, the addition of Er_2_O_3_ and Yb_2_O_3_ to dope the modified glass enables complementary active applications such as optical amplifiers or infrared lasers^23^. Although this technology has been successfully applied to phosphate and borate glasses^19,20,24^, it requires great care to detail to extend it to silicate glass, with properties suitable for multiple photonic applications.

Alternative fs-laser direct-writing technologies are based on other concurrent processes, such as lattice stress, heat accumulation, or defect creation^25^. Waveguides produced by these means have reached high performance features, with refractive index contrast close to 10^–2^ and low losses on the order of 0.1 dB/cm^26–28^. These studies have in common the use of commercially available silicate or borosilicate glasses, including Schott AF45, AF37, B33 and Corning 0211, Eagle 2000, Eagle, XG, among others^12^. It is worth highlighting the work by Lapointe et al*.* in which they are able to inscribe low loss waveguides (0.027 dB/cm) in commercial soda-lime silicate Corning Gorilla glass^29^.

Here in, we present a thorough experimental study on fs-laser induced thermophoretic writing in compositionally designed silicate glasses. We have paid special attention to the factors influencing the migration process, such as the shape of fs-laser beam focal volume, the absorbed energy and the temperature gradients, which lead to the formation of a variety of microstructures. In parallel, we evaluate the performance of these structures as infrared optical waveguides.

## Experimental methods

Glass samples belong to the silicate family and have been designed to enable the production of photonic devices upon fs-laser treatment. The composition of the modified silicate glass is 15Na_2_O-15K_2_O-10BaO-60SiO_2_, in mol%, doped with 1 wt% Er_2_O_3_ and 2 wt% Yb_2_O_3_to facilitate location and alignment of the devices thanks to the green up-conversion emission of Er^3+^ pumped at 976 nm. This particular composition facilitates the diagnosis of element migration in the glass while simultaneously enables the creation of positive refractive index contrasts. The glass was obtained by the melt quenching procedure: 2 batches of 25 g of glass were prepared by mixing carbonates and SiO_2_, calcined slowly up to 800 °C in porcelain crucibles and melted at 1450 °C for 2 h. To ensure homogeneity, a final melting was performed by mixing the two previous glasses, in a Pt crucible at 1550 °C for 2 h. The refractive index of this glass is 1.516.

High-repetition-rate fibre-based femtosecond laser pulses (Satsuma HP, Amplitude Systems) are used to induce the thermophoretic writing. A thorough description of the experimental system can be found elsewhere^22,24^. For these experiments, 400 fs pulses at 1030 nm pass a 1.3 mm slit at 500 kHz frequency are focused at different depths within the glass sample using either of the following objective lenses,

- Aspheric lens (AL): numerical aperture (NA) 0.68, focal length (FL) 3.10 mm.
- Corrected objective (CO): Comar 60 × objective lens, NA = 0.85, FL = 2.91 mm, cover glass correction (0.17 mm).
- Uncorrected objective (UO): Enosa 45 × objective, NA = 0.85, FL = 3.25 mm.

Samples typically 12 mm long are scanned at 60 μm s^−1^ during the laser writing process.

Processed samples are grinded and polished until the created structures cleanly emerge laterally. Optical microscopy is performed with a Nikon Eclipse system with a 100×, 0.9 NA objective lens and 1.5 Barlow lens. Near field guided modes are measured by coupling a SMF-28 single mode fibre and imaging with a 50 × objective and an infrared camera (Goodrich SUI) at 1500, 1550, 1590 and 1640 nm. Overall losses evaluation is carried out by introducing the output power of each waveguide at 1640 nm into a photodiode sensor (Ophir PD300). Propagation losses are calculated as overall minus coupling and Fresnel losses. Local elemental composition is obtained using energy-dispersive X-ray microanalysis (Bruker AXS Quantax μ-analysis) in a scanning electron microscope (Leica S440).

## Results and discussion

In this work, we have divided results and discussion in three different sections. The first one is dedicated to the description and analysis of the common features of the produced structures. The second section consists of a thorough study of the shapes obtained at different inscription depths. Finally, the last section addresses the performance optimization of the photonic devices produced.

### Common features

**Figure 1:**
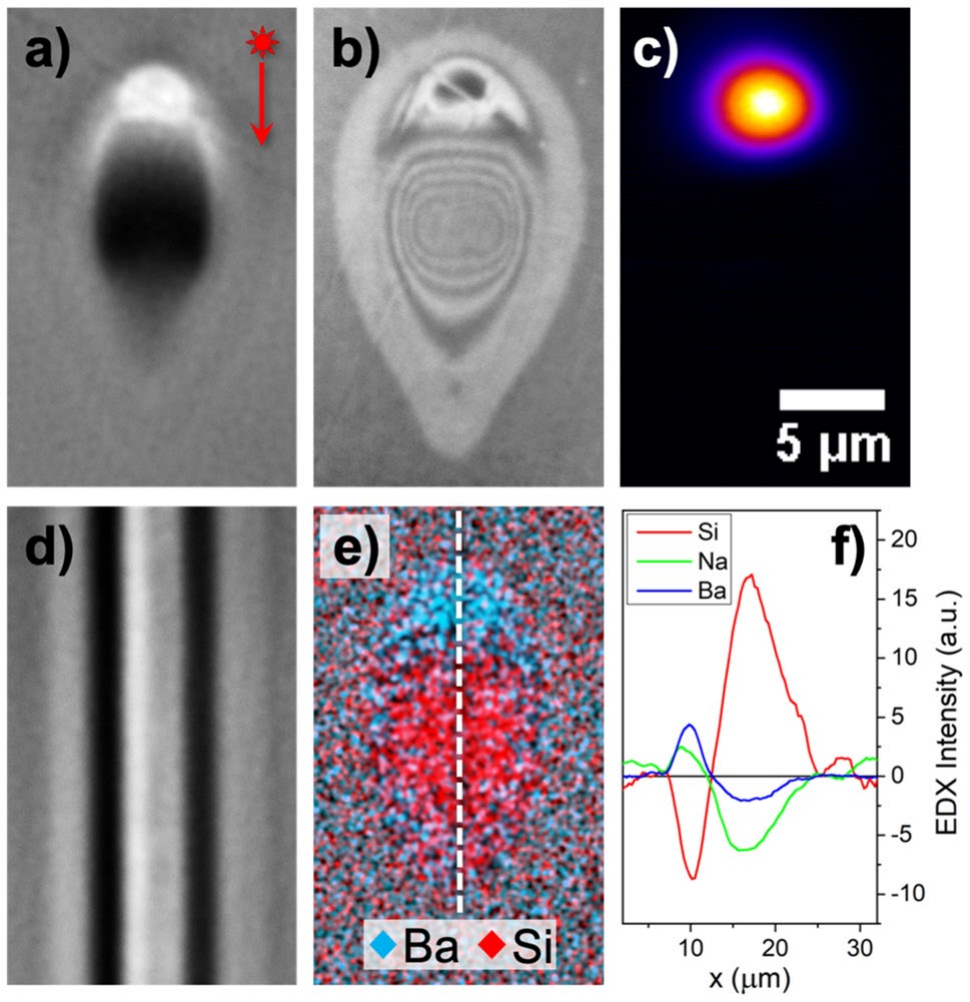
Characterization of optical waveguides produced with 530 nJ pulses and UO lens focusing at 250 µm of depth. (**a**) Transmission and (**b**) reflection cross-section optical microscopy, (**c**) near field image of the guided mode at 1640 nm, (**d**) longitudinal (top view) optical microscopy, (**e**) EDX elementary mapping and (**f**) EDX profile measured along the vertical line indicated in (**e**). Scale bar on (**c**) applies to all images. Laser beam direction is indicated by the red arrow in (**a**).

Fs-laser treatment of silicate glasses induces a local modification in the relative concentration of the material components through a thermophoresis driven phenomenon^30^. In our experiments, these modifications follow a similar pattern in the majority of the cases. To describe these common features, we have included in Fig. 1 morphological, compositional and light guiding information corresponding to irradiations using 530 nJ pulses with UO objective lens focusing at 250 μm underneath the surface. The transmission optical microscopy cross view displayed in Fig. 1a shows a well-defined bright area at the top part of the image, which suggest an increment in the local refractive index. Right below the bright spot, a more diffused but intense dark region arises, occupying an area roughly triple in size. This cross-section is consistent all along the scanning direction, as it can be seen in Fig. 1d, generating a smooth and continuous pattern throughout the width of the sample. These features are consistent with previous waveguiding structures produced in other glass matrices such as phosphates and borates^20,31^. Complementarily, a rather richer profile is found when performing optical microscopy in reflective mode (Fig. 1b). In this case, the contrast is inversed in the top part. Interestingly, the bottom part shows a ring pattern with different separations at the top, at the bottom and on the sides. These fluctuations might be mild compositional gradients that could originate from a competition between thermal diffusion (Fick’s law) and thermophoresis (Soret effect). In these circumstances, the antagonistic behavior of each driving force leads to an inverted Hopf bifurcation with instabilities arising as oscillations of increasing amplitude^32^. However, this analysis is outside the scope of this paper and will be developed in detail in a future work.

In terms of composition, EDX mapping and corresponding vertical profile included in Fig. 1e,f show the concentration of each component in the silicate glass. The general tendency observed is that the dark area appearing in optical microscopy presents a considerable enrichment in Si, while Ba, Na and K relative content reduces. On the upper side, the trend is quite the opposite, having an incremental concentration for Ba and Na, negligible variation in K and a decrease in Si. The sizes of the Si-rich and Si-poor areas observed in the EDX mapping (Fig. 1e) are consistent with optical microscopy area measurements. Additionally, it is worth noticing that the position of either maxima and minima coincide in both Si-rich and Si-poor areas, having a common axial crossing point (see Fig. 1f). These features are consistent with previous static irradiations described in the literature. That is the case of the work carried out by Kanehira et al*.*, where the authors were able to observe Si ions migrating towards the center of the molten region, while glass modifiers such as Na, K or Ca preferentially occupied the colder outer ring^33^. Additionally, Shimizu et al*.* used fs-laser irradiation upon a SiO_2_–CaO glass and a numerical model based on the thermo-diffusion equation to successfully link the concentration of each element with the temperature gradient^34^. Other studies in borosilicate glasses show similar results, with light and heavy elements (Na, K, Ca, Al, Zn) migrating away from the beam center, leaving there a silicon excess^5,35^. However, although Si predictably migrates to the higher temperature region, the behavior of glass modifiers under a given temperature gradient is difficult to predict since it depends on the global composition and the kind of element^14^. In particular, our glass composition contains Ba, a heavy element intended to increase the local refractive index in the light-guiding area, and a unique combination of other compounds that has not been studied previously in the literature.

Regarding the performance of the produced structures as waveguides, Fig. 1c displays a near field image obtained by coupling light at 1640 nm. This well confined mode field has a good degree of symmetry along the waveguide axis and a mean diameter of about 6 μm. Assuming a step-index waveguide, it is possible to combine near field measurements with the modified area observed by optical microscopy to obtain an estimation of the refractive index contrast (Δn) created^36^. In this case, this calculation leads to a Δn equal to 1.7 × 10^–2^. This value is comparable to the best results produced so far by fs-laser induced element redistribution in other glass matrices such as borates (1.2 × 10^–2^) or phosphates (1.4 × 10^–2^)^20,22^. The Δn reported here is also comparable to that of the best performing waveguides written by fs-laser in more common glasses such as fused silica or borosilicate^37,38^. In these latter cases, the formation mechanism involves processes such as lattice stress, heat accumulation or thermal diffusion^25^.

In spite of the good light confinement observed in the structure presented in Fig. 1, propagation losses have a great impact in their performance, reaching a value of 7 dB cm^−1^. In the following sections, we explore different configurations and study the associated compositional and optical characteristics to further improve the performance as waveguides of the written structures.

### Shape and depth study

In this section, we have included the results of a study where we have intentionally varied the focal volume to produce significantly different structures. This has been achieved by using different types of objective lenses, as described in the experimental section, and by focusing the fs-laser beam at different depths underneath the surface. This second approach takes advantage of the increasing spherical aberration to progressively alter the symmetry of the focal volume along the propagation axis^31,39^. It is worth mentioning that even though we have used depth as a control parameter, it is possible to reproduce any structure at a given depth by varying alternative experimental parameters^22^.

**Figure 2:**
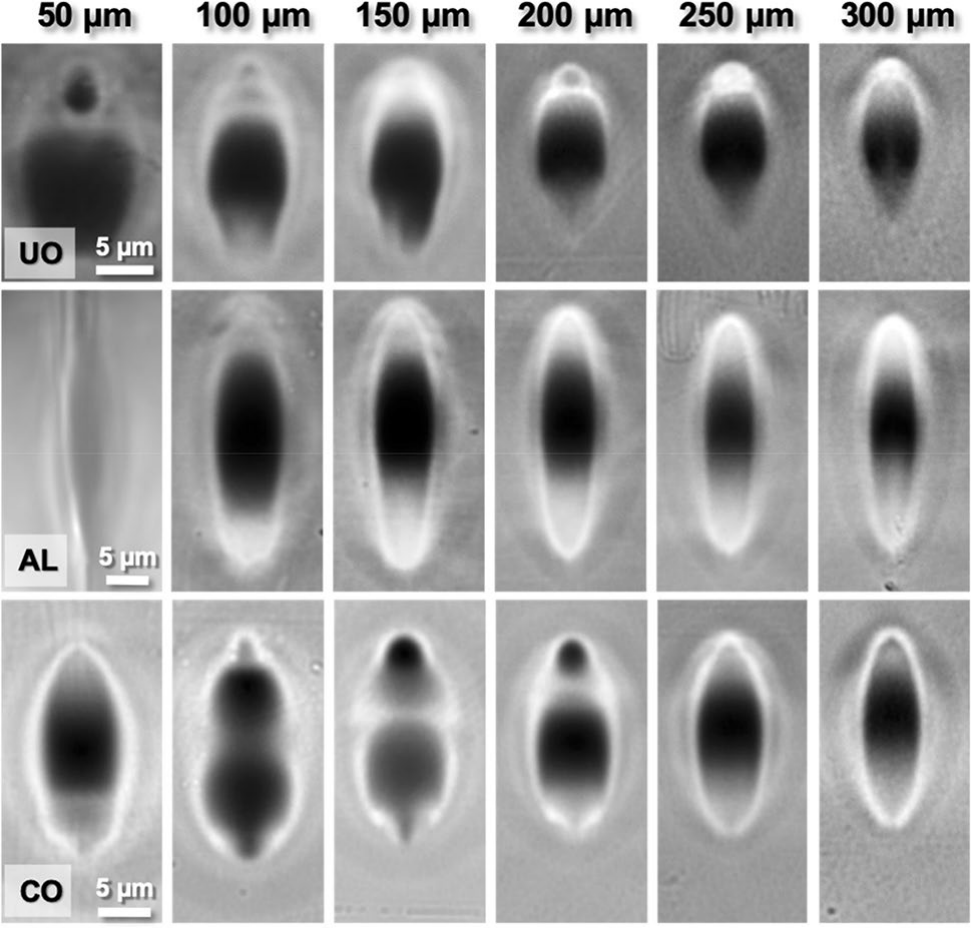
Cross-section transmission optical microscopy images of structures produced with 530 nJ pulses and objective lenses (UO, AL and CO) as labeled at the indicated depths. Scale bars apply to the images of each objective lens.

Figure 2 contains cross-section transmission optical microscopy images of structures produced using UO, AL and CO lenses focusing the fs-laser beam at depths ranging from 50 to 300 μm. At the top row of this figure, it is possible to observe that objective lens UO produces asymmetric structures with a large dark area (Si-rich) in the middle and a bright part at the top for depths greater than 100 μm. This brighter part that is associated with a positive Δn, appears as a blurry not well-defined area for low depths (up to 150 μm) and then, it begins to shrink and to present sharper edges and a brighter contrast, culminating at 250 μm of depth. For larger depths, the structures lose definition due to a combination of excessive beam elongation and low intensity caused by spherical aberration^40^. Detailed area measurements have been obtained from these images and are included in Supplementary Fig. S1 of the Supplementary Information.

The microstructures produced using AL (Fig. 2 middle row) present significant differences with respect to the previous case. As expected, the overall modified area is larger, since the NA of AL lens is lower. More significantly, produced structures show a central dark spot and two bright areas, one on top and one below. In both cases, presumably positive Δn regions are well-defined and observable at depths higher than 100 μm. In general, the area of the bottom part (AL-Bottom) is larger than the top region (AL-Top), although the ratio varies for increasing depths (see Supplementary Fig. S1 of the Supplementary Information).

Finally, CO lens has produced a wide variety of patterns depending on the focusing depth. Unlike the other lenses used in this study, CO has been designed to correct spherical aberration at 170 μm when using a typical microscopy cover slide. However, our modified silicate glass constitutes a non-matching medium and, therefore axial and off-axial light might divert forming complex patterns^41^. In the bottom row of Fig. 2, it is possible to observe CO inscriptions. At low depths (< 150 μm), Si-rich areas vary in shape and size, with narrow and poorly defined bright areas. Focusing the fs-laser beam deeper enables to progressively create larger bright areas, reaching a configuration similar to the other lenses over 250 μm. The presence of isolated dark regions at 150 and 200 μm of depth suggests that the fs-laser beam profile presents various relative maxima, leading to the production of multiple hot spots.

**Figure 3:**
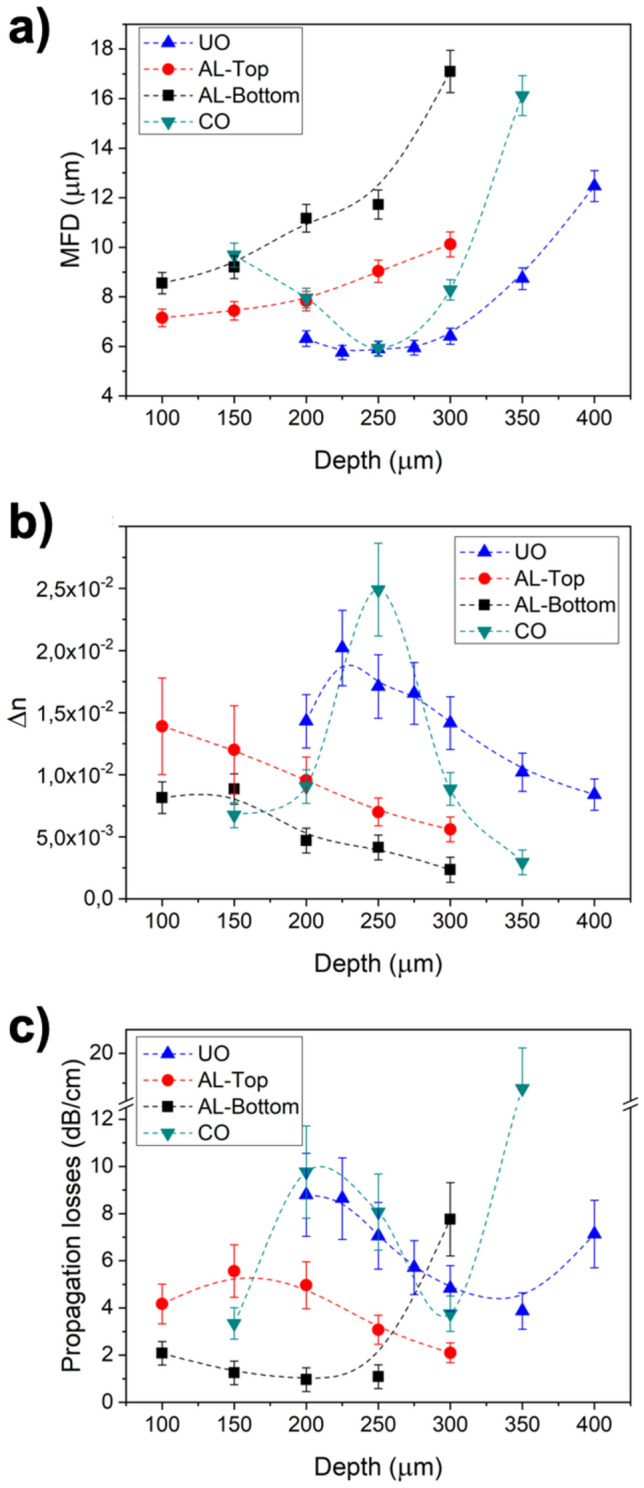
(**a**) Mode field diameter, (**b**) step refractive index and (**c**) propagation losses as a function of depth at 1640 nm for waveguides written with UO, AL and CO lenses.

The performance as waveguides of the created structures has been evaluated and its outcome is shown in Fig. 3. Although some structures were poorly guiding, the vast majority of them present efficient waveguiding characteristics (see Supplementary Fig. S2 of the Supplementary Information for near field images of the conditions studied). Mode field diameters (MFD) obtained from near field measurements at 1640 nm (Fig. 3a) span almost continuously in the 5.5–17 μm range. Particularly interesting are UO waveguides written from 200 to 300 μm deep with MFDs below 6 μm, since this field size could enable the creation of devices to successfully communicate between different photonic technologies, such as optic fibers and silicon on insulator^42^. Regarding the structures produced using the aspheric lens, both AL-Top and AL-Bottom are viable as waveguides and show the same tendency in the MFD with respect to the inscription depth, with a slightly more compressed field in AL-Top. Complementarily, CO waveguides present a wide MFD range with a minimum value of approximately 6 μm at 250 μm of depth. The low MFD achieved is significant since it could enable the fabrication of coupling devices to match the needs of the main the main technological platforms for photonic integrated circuits, that is, silica-on-silicon (SOS) and silicon-on-insulator (SOI)^25,43^.

Following the procedure indicated in the previous section, it is possible to obtain Δn for each waveguide from near field measurements^36^. These values are displayed in Fig. 3b and present diverse tendencies depending on the objective lens used. On one hand, UO exhibits Δn values from 1.5 × 10^–2^ to 2 × 10^–2^ at depths ranging from 200 to 300 μm. As commented before, these values are comparable to the best ones obtained in alternative glass matrices and with other techniques^20,22,37^. On the other hand, CO waveguides present relatively low Δn values except at 250 μm of depth, where it peaks to 2.5 × 10^–2^. Finally, AL waveguides have lower Δn values that decrease for increasing depths. In particular, AL-Top range is 0.5–1.4 × 10^–2^ and AL-Bottom varies from 0.2 × 10^–2^ to 0.8 × 10^–2^. Although these values might seem low in comparison with the others, they are sufficiently high to produce very efficiently performing waveguides.

In terms of propagation losses, the data retrieved is included in Fig. 3c. Despite the high Δn values obtained for UO waveguides, their performance in terms of propagation losses appears to be low, losing 5–9 dB cm^−1^. A similar case is observed for CO, where the Δn = 2.5·10^–2^ condition renders about 8 dB cm^−1^. It is only AL-Bottom waveguides that show attractive propagation losses, with values close to 1 dB cm^−1^. However, AL-Bottom structures had the lowest Δn range. We have made an experimental effort in trying to increase Δn in AL-Bottom waveguides while maintaining propagation losses low that is described in the next section of this document. Prior to that, we present the compositional and computational analyses that have led us to obtain further insights of the driving forces controlling the fs-laser induced thermophoresis in modified silicate glasses.

Compositional analysis carried out by EDX in a SEM shows similar trends as previously stated in Fig. 1f. That is, the dark area in the microscopy correspond to a Si-rich region, with lower concentration of all the modifiers, while bright ones are Si-poor and have Ba and Na enrichment. Measured values at the centre of each differential area for each objective lens are included in Table 1 (see Supplementary Fig. S3 of the Supplementary Information for full enrichment profiles). Although BaO can be considered as the refractive index carrier due to its larger size and higher mass, contributions of all the other elements are not negligible and need to be carefully considered. Recently, we have established a methodology to obtain an estimation of Δn using the electronic polarizability of each element present in the glass after fs-laser irradiation^19^. This analysis is based on the Lorentz–Lorenz relation between electronic polarizability (α_m_) and the refractive index of the medium (n)^44^.

1 $$\alpha_{m} = \frac{3}{4\pi }\frac{{V_{m} }}{{N_{A} }}\frac{{n^{2} - 1}}{{n^{2} + 2}},$$

where V_m_ is the molar volume. The α_m_ values needed for these calculations are included in Supplementary Table S1 of the Supplementary Information^45–47^. Using this method, we have obtained Δn_Pol_ values for each of the cases in Table 1. Estimations based on electronic polarizabilities are in good agreement with near field Δn results, showing values in the 1–2 × 10^–2^ range for Si-poor regions. However, the uncertainty level associated to polarizability-based results tends to be relatively high since it implies the combination of different EDX signals. Additionally, electronic polarizabilities methodology enables to further characterize the refractive index of the Si-rich area, which is not accessible through near field measurements. As expected, Δn of these dark-appearing regions is negative, reaching values down to – 5 × 10^–2^. Far from hampering the performance of the produced structures as waveguides, this lower refractive index region positively contributes to a higher degree of light confinement.

**Table 1:** Relative enrichment of each element in the modified silicate glass, step refractive index obtained via electronic polarizabilites (Δn_Pol_), via near field measurements (Δn_MFD_) and stoichiometric oxygen relative increment (Δ^S^O).

Lens	Zone	δSi (%)	δNa (%)	δK (%)	δBa (%)	Δn_Pol_ (10^–2^)	Δn_MFD_ (10^–2^)	Δ^S^O (%)
UO	Si-poor	− 8.3 ± 0.9	10.3 ± 1.1	− 1.0 ± 1.0	29.5 ± 1.3	1.2 ± 0.7	1.7 ± 0.3	− 1.7 ± 0.4
AL	Si-poor (top)	− 9.6 ± 0.9	22.5 ± 1.2	− 4.0 ± 1.2	33.0 ± 1.3	2.0 ± 1.1	2.2 ± 0.4	− 1.3 ± 0.3
AL	Si-poor (bottom)	− 5.3 ± 0.8	10.1 ± 1.1	− 2.2 ± 1.1	18.6 ± 1.1	0.9 ± 0.6	1.3 ± 0.3	2.0 ± 0.5
CO	Si-poor	− 5.1 ± 0.8	7.3 ± 1.1	− 1.3 ± 1.0	29.8 ± 1.3	1.9 ± 1.0	2.5 ± 0.4	− 1.0 ± 0.3
UO	Si-rich	24.0 ± 1.3	− 54.4 ± 0.5	− 42.5 ± 0.6	− 19.6 ± 0.8	− 3.7 ± 1.7		7.8 ± 2.0
AL	Si-rich	19.8 ± 1.2	− 48.8 ± 0.5	− 38.5 ± 0.6	− 29.8 ± 0.7	− 5.1 ± 2.4		8.0 ± 2.0
CO	Si-rich	20.7 ± 1.2	− 43.1 ± 0.6	− 28.0 ± 0.7	− 19.6 ± 0.8	− 2.3 ± 1.1		7.3 ± 1.8

Alternatively, compositional analysis shows an additional unforeseen effect in the irradiated area related to oxygen concentration. Following the same procedure we used for the other elements forming the glass, it is possible to use EDX data to determine the relative enrichment of O. In parallel, the data of the remaining components can be used to calculate the stoichiometric concentration needed in either Si-poor or S-rich regions. The difference of these two quantities divided by the O concentration of untreated regions is noted by Δ^S^O. If there is no deviation from the stoichiometric concentration, this parameter would be zero. However, calculations for the centre of each area included in Table 1 show that there are quantitative deviations (see Supplementary Fig. S3 of the Supplementary Information for full Δ^S^O profiles). On one hand, Si-rich areas present a positive Δ^S^O increment close to 8% in all the cases. On the other, Si-poor regions have in general minor negative Δ^S^O values with one exception, AL-Bottom structures having a positive 2% Δ^S^O value. This particularity is significant since these are precisely the better performing waveguides in terms of propagation losses. As it occurs in fused silica, fs-laser pulses could be producing an oxygen deficiency at the Si-poor regions of the modified silicate glass, leading to the production of color centers^48,49^. The absorption band that appears at the infrared region where the waveguiding performance is measured is typically associated to self-trapped holes and needs to be reduced in pure-silica-core optical fibers, among other devices^50^. These findings are in agreement with previous results by Kanehira et al*.*, who established a relationship between the migration of the modifiers and their weaker oxygen single-bond strength in relation to the network formers (Si–O) in static fs-laser irradiations^33^. Summarizing these considerations, the anomaly found in AL-Bottom waveguides could positively contribute to the low propagation losses measured.

**Figure 4:**
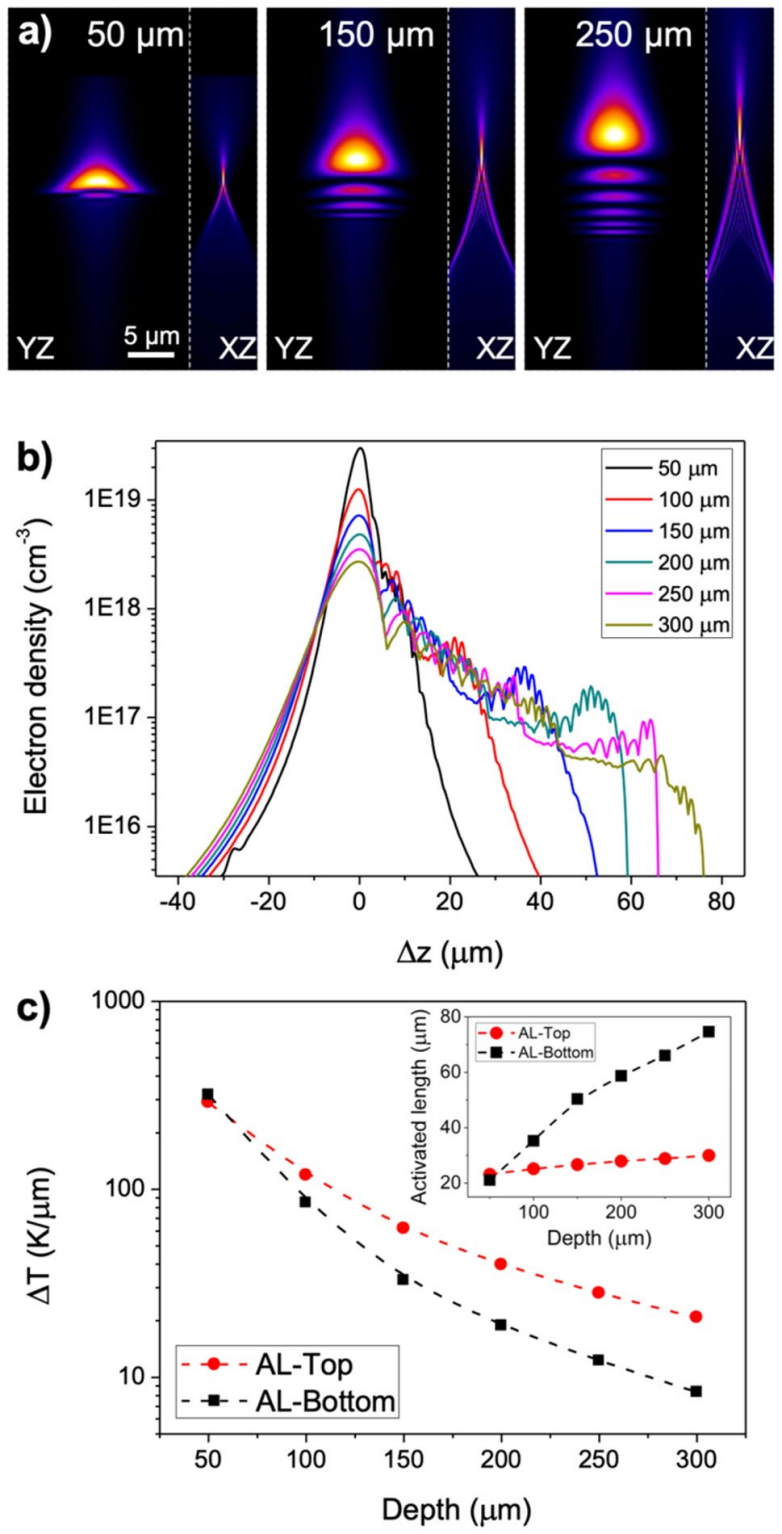
(**a**) Beam intensity for AL lens along XZ and YZ planes for the labeled focusing depths. (**b**) Electron density profiles for the labeled focusing depths. Spectra are horizontally shifted so the maximum electron density is at 0 for all the cases. (**c**) Temperature gradient (main graph) and activated length (inset) versus the focusing depth.

To further investigate fs-laser beam propagation leading to energy absorption in the irradiations carried out with AL, we have used the optical propagation simulation tool developed by del Hoyo et al*.*^51^. This tool enables to rapidly assess the nonlinear propagation of the fs-laser beam through the numerical solution of the nonlinear Schrödinger equation with a given set of processing parameters. In particular, we have assumed a nonlinear refractive index of 1 × 10^–20^ m^2^ W^−1^ and a three-photon absorption process with β_3_ = 2.7 × 10^–30^ m^3^ W^−2^ for the results shown in Fig. 4^52^. Due to the slit-shaping approach implemented in our irradiation system, the beam has a significantly different intensity pattern along the compressed (XZ) and the uncompressed (YZ) planes (beam direction has been arbitrarily associated to the Z axis). This fact is observed in Fig. 4a, where the beam intensity along the XZ and YZ is displayed for 50, 150 and 250 μm of focusing depth. Moreover, it is also possible to observe how non-linear effects tend to shift the beam towards the surface while spherical aberration elongates the beam along the Z-direction. In addition, the simulation allows to calculate the absorbed energy and to obtain an estimation of the electron density generated by multiphoton absorption. Integrating the electron density along the sample scanning direction (X-axis), we obtained the profiles included in Fig. 4b for focusing depths between 50 and 300 μm. For the sake of comparison, these electron density profiles have been shifted horizontally so their maxima coincide. As expected, we find that the maximum value of the electron density decreases when the depth is incremented. A more interesting feature is observed on each side of the maxima. On the left side (AL-Top), the slope of the curve presents an identical behavior for all depths simulated. On the right side (AL-Bottom area), we find a different tendency. In this case, curves vary their shape considerably, presenting longer electron density tails for deeper inscriptions. This implies that the forces driving the migration process in AL-Bottom are softer and apply to longer distances than in the upper neighboring region (AL-top). Although still effective, this process would be less extreme and could contribute to a better ion accommodation and therefore to the reduction of propagation losses observed in Fig. 3c.

To quantify the temperature gradient, we considered a three-photon thermodynamically adiabatic process and the electron density profiles in Fig. 4b^13^. In addition, we need to consider the heat capacity (c_p_ = 0.84 Jg^−1^ K^−1^) and the density (ρ = 2940 kg m^−3^) of our glass. The results displayed in Fig. 4c show great temperature gradients (up to 300 K μm^−1^) on both sides of the maximum at low irradiation depths. However, the differences between both sides of the maximum are clearly noticeable for treatments deeper than 100 μm, where the gradient on AL-Top is typically twice that on AL-Bottom. Simultaneously, the activated length under the temperature gradient for AL-Bottom turns out to be much greater, which increases the competition among migrant species (see Fig. 3c inset). The values obtained for the temperature gradient are consistent with previous data retrieved in static fs-laser irradiations in silicates. That is the case of Shimizu et al., that using 80 fs pulses at 250 kHz repetition rate with 1 μJ of energy and a NA = 0.4 objective lens upon a soda lime glass produced gradients of up to 200 K μm^−1^^8^. Similarly, results published by Miyamoto et al*.* show temperature gradients of 280 K μm^−1^ under similar irradiation conditions (10 ps pulses, 50 kHz, 10.3 μJ, NA = 0.55, borosilicate glass)^18^. These temperature gradients might seem extremely high when compared with classical thermophoresis studies in liquid silicates or basalt magma where typical values are on the order of 0.05 K μm^−1^^53,54^. However, there is an even greater difference in terms of time scale. While thermal diffusion in naturally occurring environments last for days^54^, fs-laser induced thermophoresis strikes are shorter than a second^17^.

Similarly, we have used the optical propagation simulation tool to assess the nonlinear propagation of the fs-laser for irradiations using CO and UO objectives. In particular, simulated electron densities for a number of focusing depths ranging from 50 to 250 μm are included in Supplementary Figs. S4 and S5 of the Supplementary Information. The particular design of CO objective that corrects spherical aberration at 170 μm of depth renders in an amalgam of complex patterns (Supplementary Fig. S4 of the Supplementary Information). These patterns present not one, as it is the case of AL lens, but multiple maxima in the order of 10^19^ cm^−3^ and whose distribution notably vary with the focusing depth. Such distributions agree with the microscopic modifications presented in Fig. 2, where dark (Si-rich) and clear (Si-poor) areas intercalate as a consequence of the multiple temperature gradients induced. Regarding UO objective, a single maximum is observed in each focusing depth, with maximum electron densities ranging from 7 × 10^19^ to 1 × 10^17^ cm^−3^. The distribution loses intensity and symmetry as spherical aberration becomes more acute for higher depths, supporting the patterns observed by optical microscopy (Fig. 2).

So far, the results described in the previous paragraphs provide an insight on the influence of a temperature gradient on the produced structure in terms of elemental composition and performance as photonic devices. Nonetheless, thermophoresis driven migration requires not only a gradient, but a minimum activation temperature^55^. Previous studies relate the threshold temperature for thermophoretic migration in silicate glasses to a viscosity level of 10^8^ Pa s^56^. In the next section, we evaluate the influence of this parameter on the morphology and performance of the written structures.

### Losses optimization

To assess the influence of the activation temperature, we have performed a series of irradiations with increasing energy at a fixed depth (150 μm) using AL as objective lens. Fixing the focusing depth enables to maintain a similar temperature gradient in all irradiations, easing the evaluation of the effect produced by an increasing energy. Additionally, reasons to employ AL objective lens are two folded. It enables to produce twice the number of waveguiding structures than the other objective lenses in this study, and it further expands the knowledge on AL-Bottom region, the most promising one in terms of propagation losses.

**Figure 5:**
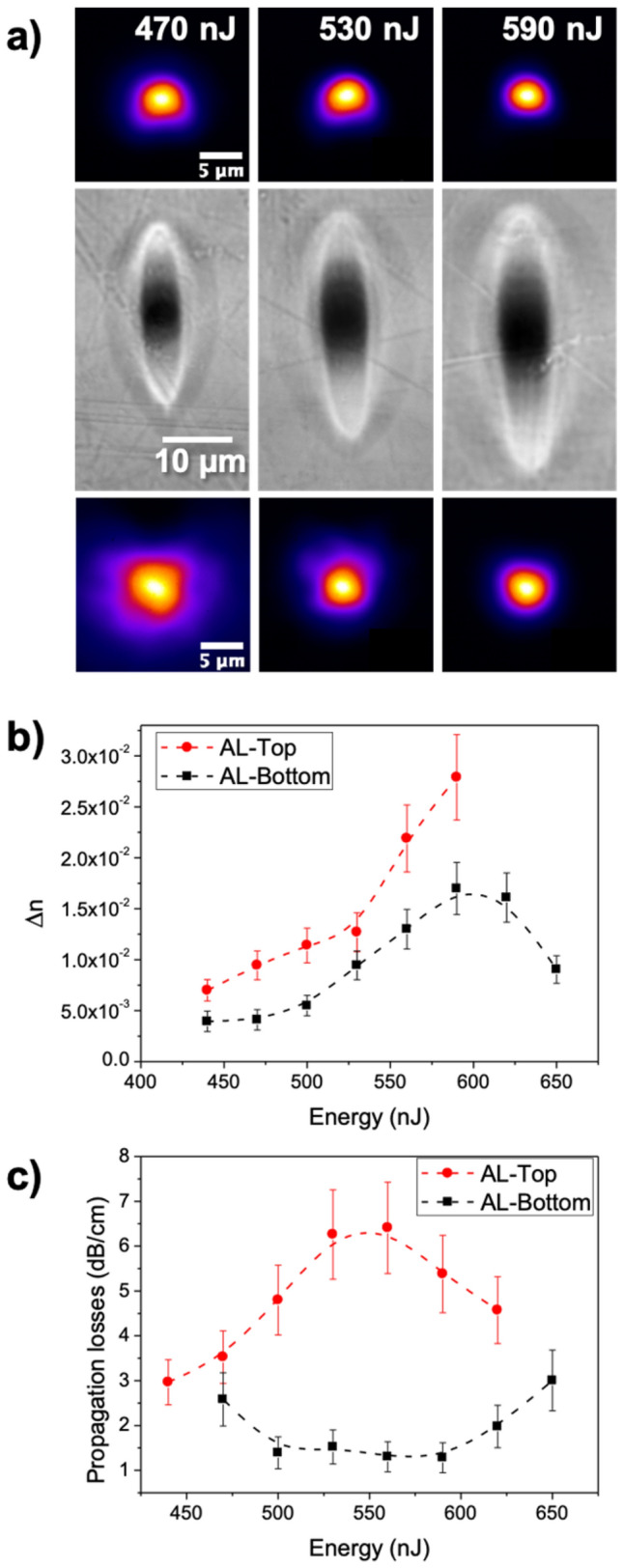
(**a**) Mode fields (top and bottom panels) and optical transmission microscopy (middle panel) of irradiations at the indicated pulse energies, (**b**) step refractive index and (**c**) propagation losses as a function of pulse energy at 1640 nm for waveguides written with AL objective lens.

Figure 5 presents a summary of the structures produced with pulse energies ranging from 440 to 650 nJ and their performance as waveguides. Optical microscopy (Fig. 5a) shows a similar shape as it was observed in Fig. 2 for AL inscriptions. The dependence of the size on either AL-Top or AL-Bottom seems to be the same, the area of the bright region increases for higher energies (see Supplementary Fig. S6 of Supplementary Information). This growth of the positive Δn area agrees with previous studies in other glass matrices^20,22^. Within the energy range of this study, there are negligible variations in the beam intensity profile. Therefore, the energy difference translates into an increment of the peak temperature, which consequently increases the size of the area with temperatures above the activation threshold^18^.

In terms of characterization as waveguides, near field measurements have been carried out. As displayed in Fig. 5a, the MFD decreases with increasing energies for both AL-Top and AL-Bottom waveguides (see Supplementary Fig. S6 of Supplementary Information for full MFD measurements). Actually, for energies equal or greater than 620 nJ, the field emitted by AL-Top waveguides becomes multimode. To calculate the associated Δn in the remaining structures presenting monomodal field distributions, we have followed the same procedure as indicated in the previous section^36^, obtaining the results included in Fig. 5b. In general, results show that increasing the pulse energy produces structures with higher Δn, ranging from 0.7 × 10^–2^ to 2.7 × 10^–2^ for AL-top and from 0.4 × 10^–2^ to 1.5 × 10^–2^ for AL-Bottom. However, there is an apparent limit for the second type, since Δn decreases from 620 nJ and above. At this point, the modified area might be exceedingly large, disabling an effective migration of the glass modifiers. Regarding propagation losses, collected data is shown in Fig. 5c. As it happened in the depth-resolved study, AL-Top presents significantly higher losses than AL-Bottom, above 3 dB cm^−1^ in all the cases. Alternatively, AL-Bottom waveguides present losses of the order of 1 dB cm^−1^ in the energy range 500–590 nJ. This energy range is large enough to produce a Δn of up to 1.5·10^–2^ while keeping losses at a low level (~ 1 dB cm^−1^). This conjunction of properties makes AL-Bottom waveguides suitable for implementation in diverse photonic devices, especially in those dedicated to infrared optical communications C-band^57^. Additionally, Er_2_O_3_ and Yb_2_O_3_ doping included in our modified silicate glass enables further active applications such as optical amplifiers or infrared lasers^23^.

## Conclusion

In summary, we have studied the fs-laser induced thermophoretic element redistribution in modified silicate glasses. We have analyzed the influence of the focal volume on the compositional redistributions and the performance of the microstructures as optical waveguides. Near field measurements and electronic polarizabilities analysis demonstrate that the guiding region presents a positive Δn that can reach values clearly above 10^–2^. Through beam propagation simulations, we have been able to establish a relationship between light propagation properties and the temperature gradient controlling the thermophoresis when the glass is in a viscous regime. Finally, we have optimized the functionality of the microstructures as waveguides with an adjustable Δn (0.5–1.5 × 10^–2^) while keeping propagation losses low (~ 1 dB cm^−1^).

## Supplementary Information

Supplementary Information.
