# Supplementary material for: Femtosecond laser induced thermophoretic writing of waveguides in silicate glass
Source: Sci Rep. 2021 Apr 16;11:8390. doi: 10.1038/s41598-021-87765-z (PMC8052338; doi:10.1038/s41598-021-87765-z)
Supplement: Supplementary file 1 — Supplementary Information. [file 41598_2021_87765_MOESM1_ESM.pdf]

Supporting Information

Femtosecond laser induced thermophoretic writing of waveguides in silicate glass

Manuel Macias-Montero,[1] Francisco Muñoz,[2] Belén Sotillo,[3] Jesús del Hoyo,[4] Rocío Ariza,[1,3] Paloma Fernandez,[3] Jan Siegel,[1] and Javier Solis[1]

[1]Laser Processing Group, Institute of Optics (IO, CSIC), Serrano 121, Madrid, 28006, Spain.

[2]Institute of Ceramics and Glass (ICV, CSIC), Kelsen 5, Madrid, 28049, Spain.

[3]Department of Materials Physics, Faculty of Physics, University Complutense of Madrid, Madrid, 28040, Spain.

[4]Department of Optics, Faculty of Physics, University Complutense of Madrid, Madrid, 28040, Spain.

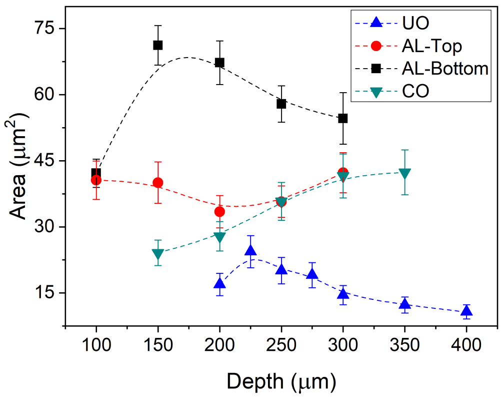

Figure S1. Area of the guiding region vs. focusing depth for the indicated objective lenses and sub-regions.

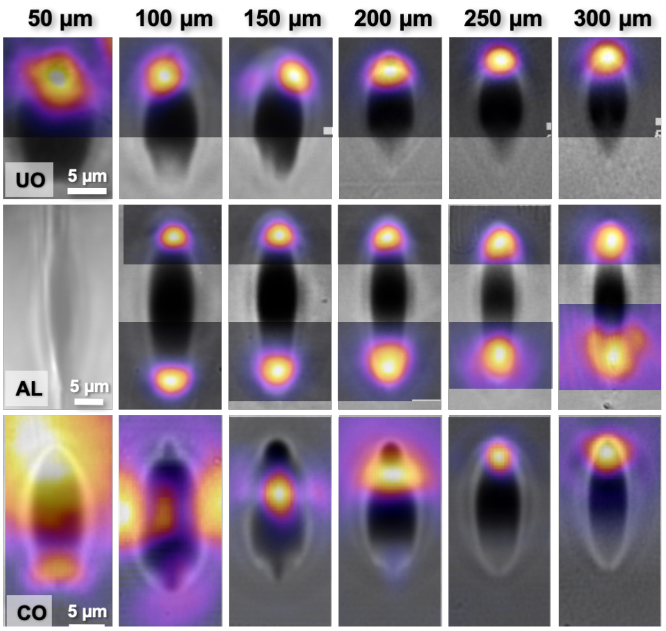

Figure S2. Near field images combined with cross-section transmission optical microscopy images for structures produced with 530 nJ pulses and objective lenses (UO, AL and CO) as labelled at the indicated depths. Scale bars apply to the images of each objective lens.

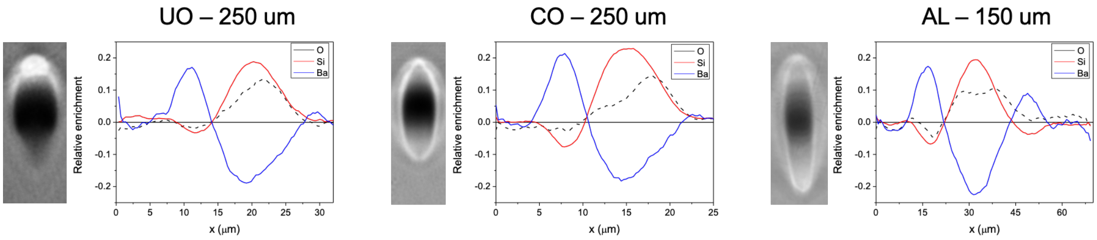

Figure S3. Optical microscopy and EDX enrichment profiles for the indicated objective lenses.

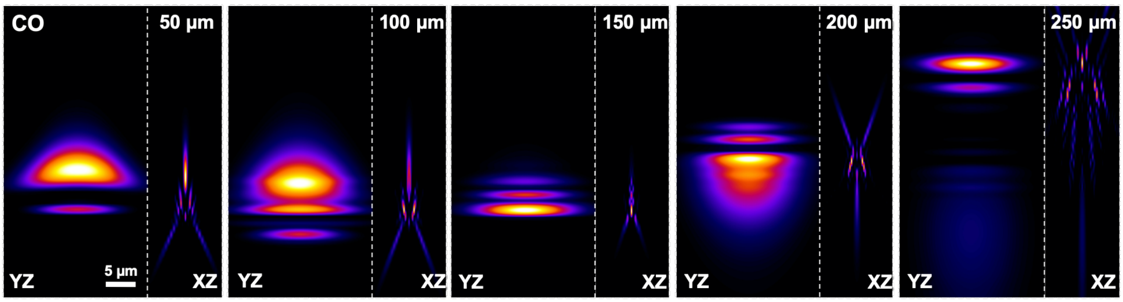

Figure S4. Simulated electron density for CO objective along XZ and YZ planes for the labeled focusing depths.

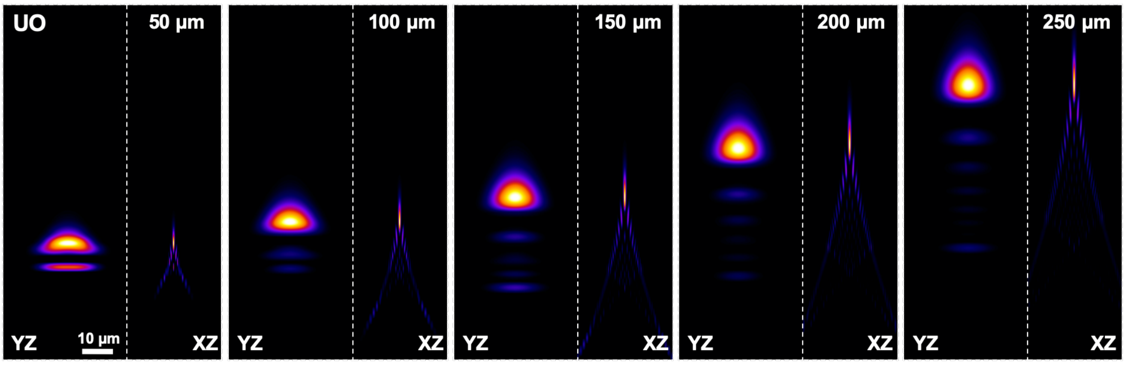

Figure S5. Simulated electron density for UO objective along XZ and YZ planes for the labeled focusing depths.

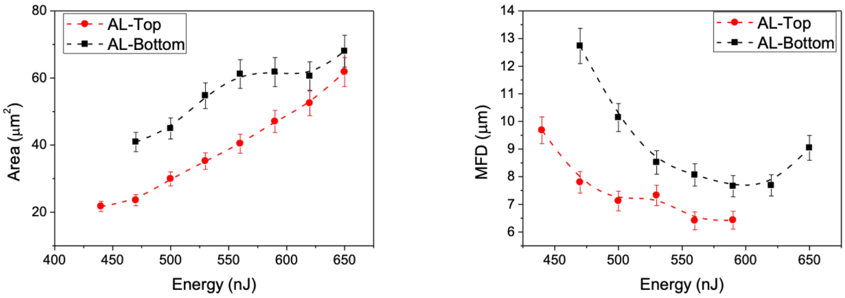

Figure S6. Area of the guiding region (left panel) and mode field diameter (right panel) vs. pulse energy for waveguides produced using AL objective lens.

Table S1. Electronic polarizabilities of the main oxide components in the modified silicate glass.

Polarizability (Å^3)	SiO_2	Na_2O	K_2O	BaO
α_{cation}	0.033	0.175	0.841	1.595
α_O^{2-}	1.427	3.221	1.858	3.652
α_{Total}	2.887	3.571	3.540	5.247
